# Standardized Fraction of *Turbinaria ornata* Alleviates Dextran Sulfate Sodium-Induced Chronic Colitis in C57BL/6 Mice via Upregulation of FOXP3^+^ Regulatory T Cells

**DOI:** 10.3390/biom10101463

**Published:** 2020-10-20

**Authors:** Na-Hyun Kim, Seon Min Lee, Yun Na Kim, You-Jin Jeon, Jeong-Doo Heo, Eun Ju Jeong, Jung-Rae Rho

**Affiliations:** 1 Gyeongnam Department of Environment & Toxicology, Korea Institute of Toxicology, 17 Jegok-gil, Munsan-eup 52834, Korea; nhkim@kitox.re.kr (N.-H.K.); smlee84@kitox.re.kr (S.M.L.); jdher@kitox.re.kr (J.-D.H.); 2 Department of Agronomy and Medicinal Plant Resources, Gyeongnam National University of Science and Technology, Jinju 52725, Korea; skdbssk@naver.com; 3 Department of Marine Life Sciences, Jeju National University, Jeju 63243, Korea; youjin2014@gmail.com; 4 Department of Oceanography, Kunsan National University, Kunsan 54150, Korea

**Keywords:** *Turbinaria ornata*, brown algae, ulcerative colitis, dextran sulfate sodium-induced chronic colitis, regulatory T cell, interleukin-10, tumor necrosis factor-α, phosphorylated signal transducer and activator of transcription-3

## Abstract

*Turbinaria ornata* is a tropical brown algae (seaweed) known to have anti-inflammatory properties. In this study, we analyzed *T. ornata* extract (TOE) using liquid chromatography quadrupole time of flight mass spectrometry (LC-QTOF-MS) and nuclear magnetic resonance (NMR) and evaluated the in vivo efficacy of TOE against dextran sulfate sodium-induced chronic colitis in C57BL/6 mice. The bioactive fraction of TOE was administered orally daily for 6 weeks to mice under different treatments normal, colitis, and colitis + conventional drug (5-aminosalicylic acid, 5-ASA). Regarding clinical manifestation, the disease activity index and colon length of the colitis + TOE group were significantly reduced compared to those of the colitis group. The results of myeloperoxidase activity and histopathological examination showed similar results. Western blot analysis of colon tissues revealed that cyclooxygenase-2, tumor necrosis factor alpha (TNF-α), and phosphorylated signal transducer and activator of transcription-3 (p-STAT3) were significantly decreased in the colitis + 5-ASA group, whereas forkhead box P3 (FOXP3) was increased. qPCR results showed changes in T cell subsets; the administration of TOE upregulated regulatory T cell (Treg) expression, although T helper 17 cell (Th17) expression did not change significantly. Interestingly, the colitis + TOE group showed high levels of both Th1 and Th2 transcription factors, but the secreted cytokine interferon (IFN)-γ and interleukin (IL)-4 remained unchanged and somewhat reduced. Additionally, TNF-α gene expression was significantly reduced in the colitis + TOE group. IL-6 mRNA levels were also decreased, although not significantly. Four compounds were structurally elucidated using 1D- and 2D-NMR spectroscopy, and five compounds were fully identified or tentatively characterized using LC-QTOF-MS. In conclusion, TOE could alleviate chronic colitis via upregulation of Foxp3^+^ Treg cells and production of the anti-inflammatory cytokine IL-10, which directly inhibits macrophages and pro-inflammatory cytokine synthesis, leading to reduced colitis.

## 1. Introduction

Ulcerative colitis (UC) is a type of inflammatory bowel disease (IBD), which is an idiopathic, chronic obstructive disease with typical recurrent episodes of colitis symptoms [1,2]. Recently, it has been acknowledged that UC mainly affects young adults (15 to 25 years old) in western countries, and its prevalence has increased over the years with no effective cure [3,4]. Although the pathogenesis of UC is not yet fully understood, many factors have been suggested as possible causes of UC, such as genetic abnormalities, environmental stresses, gut microbiota, and immune disorders [5].

IBD is known to initiate when the intestinal epithelial barrier is broken down by toxic chemicals, pathogens, or idiopathic causes [6]. Once this barrier collapses, the underlying tissues are exposed and attacked by gut microbiota, leading to the recruitment of leukocytes into the lamina propria and causing uncontrolled inflammation [6]. During the progression of UC, activated macrophages produce pro-inflammatory cytokines such as tumor necrosis factor alpha (TNF-α), interleukin (IL)-1 β, IL-6, and IL-12 which attract neutrophils to the colonic mucosa, and directly contribute to crypt abscess and ulcerative lesions by producing reactive oxygen species and proteolytic enzymes [7,8]. Particularly, TNF-α is a major pathological cytokine that can affect the activation and progression of colitis by including direct disruption of the intestinal epithelial barrier integrity, provocation of epithelial cell death, and co-stimulation of effector T cells toward further chronic inflammation [9,10].

It was also revealed that IBD has been associated with exaggerated cluster of differentiation 4 (CD4)^+^ T cell response [11]. In the normal healthy state, CD4^+^ T cell subsets exist in homeostasis so that they can resolve pathogens and inflammation [12]. However, under IBD conditions, these normal processes are disrupted [12]. The Th1 subset, differentiated by IL-12 stimulation, expression of transcription factor T-bet, and release of interferon (IFN)-γ, IL-6, and IL-12 is predominant in Crohn’s disease (CD), whereas Th2, influenced by IL-4 or IL-5, is dependent on GATA binding protein 3 (GATA3) and its secreted cytokines IL-4, IL-5, and IL-13 are activated in UC. In addition, both CD and UC are commonly exhibited in Th17 subsets that are differentiated by the transcription factor retinoic acid-related orphan receptor γt (RORγt) and produce IL-17, IL-21, and IL-22, whereas the forkhead box P3 (FOXP3)-induced regulatory T cell (Treg) subset is decreased [13,14,15]. Many controversies remain over how these immune imbalances work on UC [16]; however, the investigation of these immune responses is important to lead toward a new UC drug discovery.

Regarding signal pathways, signal transducer and activator of transcription-3 (STAT3) is known to play a central role in IBD. It is widely expressed in various cell types, such as T cells, macrophages, and epithelial cells, and is activated mainly by IL-6 and leads to chronic inflammation [17]. In a recent study, STAT3 was activated in many patients with IBD and increased STAT3 was directly correlated with the severity of colitis and contributed substantially to colitis [17,18].

Conventional drugs that are clinically applied to patients with UC include anti-inflammatory agents such as corticosteroids and 5-aminosalicylic acid (5-ASA), immunosuppressants (e.g., azathioprine), and biological agents (e.g., infliximab) [19]. The 5-ASA is used as a first-line treatment for patients with mild and moderate UC owing to fewer side effects than those with other drugs. However, most of these are not adequate for long-term administration and/or cause significant side effects [20]. In these circumstances, a search for improved therapeutic agents is essential [21]. Naturally derived compounds have long been used in treating human diseases. The superiority of natural products as drugs is derived from their structural diversity and complexity. Owing to the chemical complexity of natural products, they often tend to affect one or more targets, thus allowing more pathways to be influenced [22].

*Turbinaria ornata* is a tropical brown alga native to the South Pacific. To date, secondary metabolites, including cytotoxic secosqualene carboxylic acid and turbinaric acid, and phenolics with antioxidant and antibacterial activity have been reported from *T. ornata* [23,24,25,26] Recently, the anti-inflammatory activity of *T. ornata* containing fucoidan as the active constituent has been reported [27]. As a part of further studies to determine the anti-inflammatory potential of *T. ornata*, our research group newly identified bioactive metabolites, sulfoquinovosyl monoacylglycerols, which regulate intestinal inflammation in vitro [28]. It was found that the butanolic fraction of *T. ornata* extract and sulfoquinovosyl monoacylglycerols markedly downregulated inflammatory mediators in response to inhibition of nuclear factor kappa-light-chain-enhancer of activated B cells (NF-κB) translocation to the nucleus in the Caco-2 and THP-1 co-culture system. Based on the results of the in vitro study, we attempted to evaluate the anti-inflammatory potential of the fraction of *T. ornata* extract in vivo.

For in vivo studies of UC, we used dextran sulfate sodium (DSS) which is widely used as a colitis inducer [29]. It is well known that colitis induced by DSS in murine models exhibits pathological features similar to those of human patients with UC—ulceration of the mucosal layer, bloody stool, and persistence of inflammatory cell infiltration in the distal colon [30]. In practice, treatment of three to five repeated cycles of low concentrations of DSS in mice is regarded as an effective and reproducible experimental method for chronic relapsing colitis [31]. Thus, a UC-like chronic relapsing colitis mouse model was induced with DSS, and the effects of the active fraction of *T. ornata* extract (TOE) were examined.

The therapeutic effects of TOE administration were compared to those of the conventional anti-inflammatory drug 5-ASA, and the underlying anti-inflammatory mechanisms associated with CD4^+^ T cell subsets were explored. Specifically, the disease activity index (DAI), colon length, and histopathology were used to evaluate clinical manifestations. Myeloperoxidase (MPO), cyclooxygenase-2 (COX-2), and TNF-α were selected for evaluating the overall inflammatory response. CD4^+^ T cell subsets in the colitis tissues were identified using qPCR—*T-bet* and *Ifn-γ* for Th1, *Gata-3* and *Il-4* for Th2, *Rorγt* and *Il-17* for Th17, and *Foxp3* and *Il-10* for Treg subsets. *Tnf-α* and *Il-6* mRNA expression levels were also examined to assess macrophage and monocyte activity. Phosphorylated STAT3 (p-STAT3) was also examined by western blotting. In addition, the main constituents present in TOE that could be responsible for its pharmacological effects were identified using liquid chromatography quadrupole time of flight mass spectrometry (LC-QTOF-MS) analysis.

## 2. Results

### 2.1. Isolation and Structure Elucidation of Compounds ***1**, **5**–**7*** from TOE

**Figure 1:**
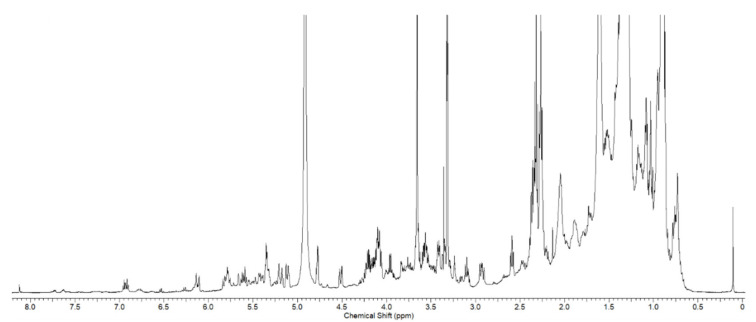
^1^H NMR spectrum of the M2 fraction of *Turbinaria ornata* extract (TOE) in CD_3_OD (500 MHz for ^1^H).

**Figure 2:**
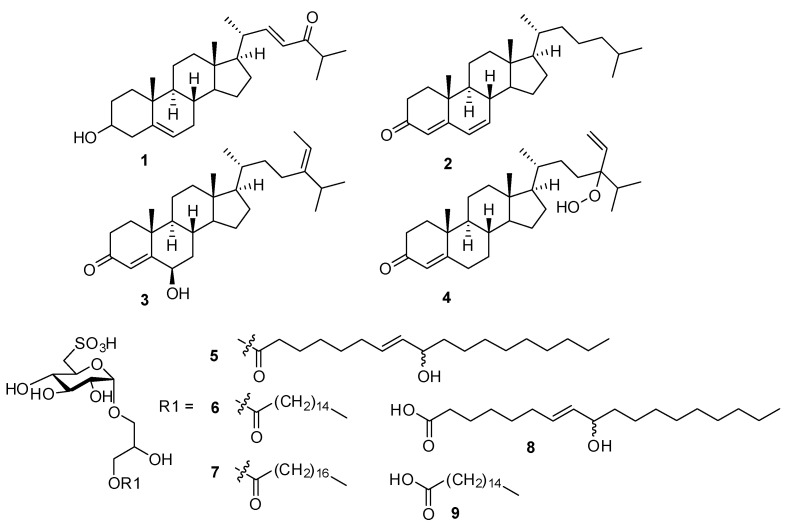
Structures of compounds **1**–**9** identified by NMR and LC-MS analysis from the M2 fraction of *Turbinaria ornata* extract (TOE).

*T. ornata* was extracted with 90% aq. EtOH and fractioned using Sephadex LH20 into four fractions (M1–M4). Each fraction was evaluated for inhibitory activity against pro-inflammatory cytokines in vitro (Supplementary Materials S1). The M2 fraction, which exhibited good activity, was selected for further in vivo tests. The ^1^H nuclear magnetic resonance (NMR) spectrum of M2 is shown in Figure 1, which contains severely overlapped signals in the upfield and oxygen-bearing regions. The separation of the main components from the M2 fraction was performed by high-performance liquid chromatography. The isolated compounds (**1**–**9**) were found to be steroids and sulfoquinovosyl monoacylglycerols (Figure 2). Using the combination of NMR and high resolution electrospray ionisation mass spectrometry (HRESIMS), and comparing with reported data, **1** was identified as 22*E*-3β-cholesta-5,22-dien-24-one [32]. The structure of **5** was determined to be 1-*O*-[(7*E*)-9-hydroxyoctadeca-7-enoyl]-3-*O*-(6-sulfo-α-d-quinovopyranosyl)-sn-glycerol, **6** as 1-palmitoyl-3-*O*-(6-sulfo-α-d-quinovopyranosyl)-sn-glycerol, and **7** as 1-stearoyl-3-*O*-(6-sulfo-α-d-quinovopyranosyl)-sn-glycerol [28,33,34].

### 2.2. LC-QTOF-MS Analysis of T. ornata

**Figure 3:**
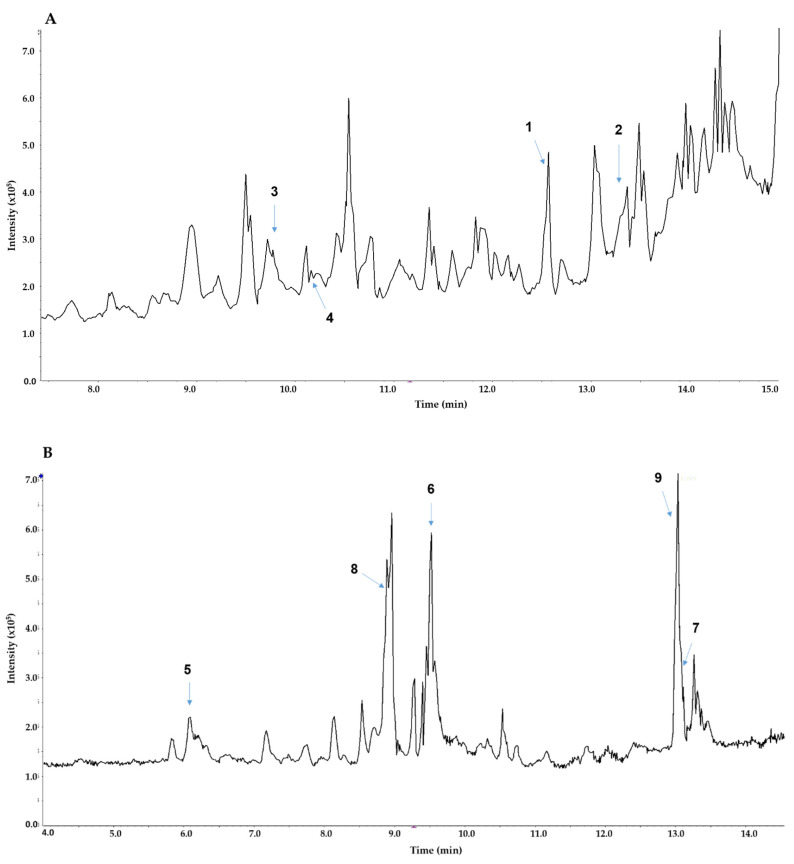
Total ion chromatogram and key peak identification of the M2 fraction of *Turbinaria ornata* extract (TOE) in positive mode (**A**) and negative mode (**B**).

Based on the information on compounds isolated from TOE, LC-MS analysis was performed. LC-MS analysis enabled accurate identification of nine compounds, including those reported from *Turbinaria* sp., based on the MS and MS/MS fragmentation data. In the total ion chromatogram of the positive ion mode, cholesta-4,6-dien-3-one (**2**), and two steroids (**3** and **4**) reported from *Turbinaria conoides* were accurately identified from the MS and MS/MS spectra [35]. In the negative ESI chromatogram, three sulfoquinovosyl monoacylglycerols were observed with two main components. The first one was characterized as a sulfoquinovosyl monoacylglycerol with 9-hydroxyoctadec-7-enoic acid (**8**) based on accurate MS and MS/MS fragmentation data at *m*/*z* 155 and 171. Similarly, the other was identified as palmitic acid (**9**) (Figure 3). The retention time and characteristic fragment ions of all identified components (**1**–**9**) are presented in Table 1.

### 2.3. Clinical Manifestation of Relief from Chronic Colitis Owing to TOE

**Figure 4:**
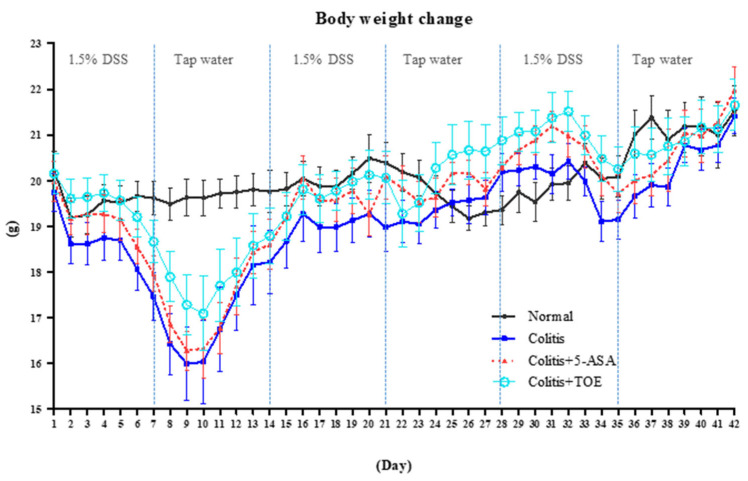
Effect of M2 fraction of *Turbinaria ornata* extract (TOE) on body weight in C57BL/6 mice with dextran sulfate sodium (DSS)-induced chronic colitis. The body weight of each mouse was measured daily. The values are presented as the mean body weight of each group ± SD. Normal, DSS-/D.W; colitis, DSS + /D.W, colitis + 5-aminosalicylic acid (5-ASA), DSS + /5-ASA 50 mg per kg; colitis + TOE, DSS + /TOE 15 mg per kg.

**Figure 5:**
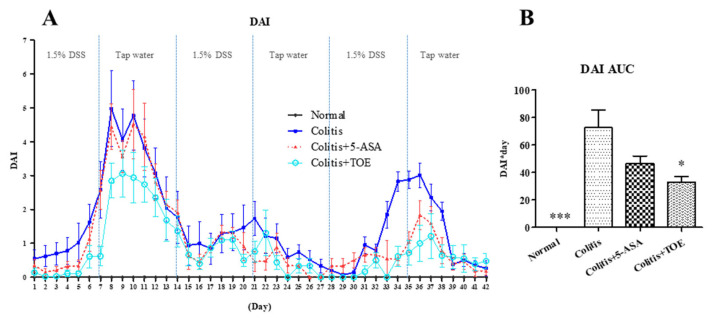
Effect of M2 fraction of *Turbinaria ornata* extract (TOE) on clinical severity of DSS-induced chronic colitis in C57BL/6 mice. (**A**) Disease activity index (DAI) score (**B**) DAI area under the curve (AUC) graph. * *p* < 0.05, *** *p* < 0.001, compared with the colitis group. Normal, DSS-/D.W; Colitis, DSS + /D.W, Colitis + 5-ASA, DSS + /5-ASA 50 mg per kg; Colitis + TOE, DSS + /TOE 15 mg per kg.

The effectiveness of the standardized M2 fraction of *T. ornata* (TOE) against chronic colitis was evaluated in DSS-induced C57BL/6 mice. All experimental animals were observed carefully for their clinical signs and weighed daily for 6 weeks. The mean body weight of the colitis induction groups decreased sharply in the first cycle of DSS + water supply. Contrary to our expectations, both clinical signs and degree of weight loss of the DSS + treatment groups (colitis, colitis + 5-ASA, colitis + TOE groups) were minimized in the second cycle, but not in the third cycle (Figure 4). The DAI graph showed three clear peaks: the first peak was the highest, the second peak was the smallest, and the third peak appeared as an intermediate value. According to our scoring system, the DAI of mice in the normal group was normalized to the zero point, so that it was not visible on the graph (Figure 5A). For a comprehensive evaluation of clinical symptoms of colitis throughout the experimental period, DAI scores were converted to DAI area under the curve (AUC). The AUC of the colitis + TOE group was lower (33.07 ± 9.91) than that of the colitis group (72.62 ± 31.48). The DAI AUC of the 5-ASA group was also lower (46.50 ± 13.44) than that of the colitis group, although the difference was not significant (Figure 5B).

### 2.4. TOE Alleviates Gross and Microscopic Chronic Colitis Lesions

**Figure 6:**
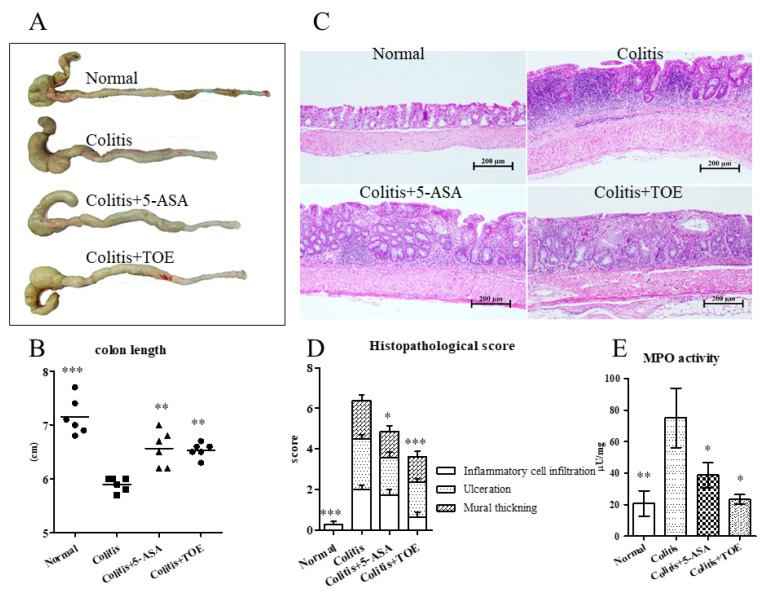
Effect of M2 fraction of *Turbinaria ornata* extract (TOE) on gross and microscopic examination of DSS-induced chronic colitis in C57BL/6 mice. (**A**) Gross lesions of colitis. (**B**) Colon length. (**C**) Microscopic examinations of hematoxylin and eosin (H&E) stained colon tissues (×10 magnification, bar = 200 µm). (**D**) Histopathological scores of colitis. (**E**) Myeloperoxidase (MPO) activity of colon tissue lysates. * *p* < 0.05, ** *p* < 0.01, *** *p* < 0.001, compared with the colitis group. Normal, DSS-/D.W; colitis, DSS + /D.W, colitis + 5-ASA, DSS + /5-ASA 50 mg per kg; colitis + TOE, DSS + /TOE 15 mg per kg.

After euthanizing all animals, the colons were quickly collected and measured. The mean length of the colon from the colitis + TOE (6.53 ± 0.14 cm) and colitis + 5-ASA (6.57 ± 0.33 cm) groups was significantly longer than that from the colitis group (5.9 ± 0.13 cm). Normal colons showed the highest value (7.15 ± 0.34 cm) (Figure 6A,B). Histopathologic examination revealed that 42 days of 1.5% DSS and water interval cycle resulted in chronic colitis lesions, showing typical abnormally proliferated epithelium with mononuclear and/or lymphoid cell infiltration, mottled fibrotic change, and mural thickening, mainly affecting the middle and distal parts of the colon. In the colitis + 5-ASA or colitis + TOE groups, the severe lesions were less common in the colons than those in the colitis group (Figure 6C). Specifically, the histopathological score (3.63 ± 1.51) in the colitis + TOE group was significantly lower than that in the colitis group (6.38 ± 1.3). The scores of the colitis + 5-ASA group (4.86 ± 1.07) also showed a similar trend. Normal healthy colons showed only minor mononuclear cells in the epithelium (0.25 ± 0.46) (Figure 6D).

### 2.5. TOE Reduced MPO Activity and COX-2 and TNF-α Expression in Chronic Colitis

**Figure 7:**
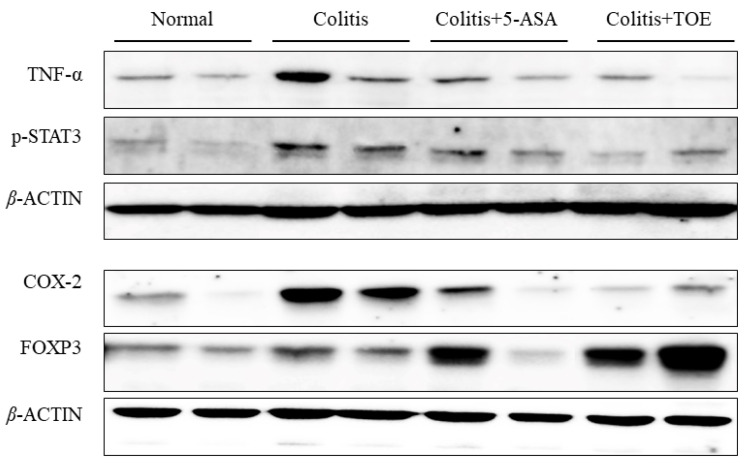
Effect of M2 fraction of *Turbinaria ornata* extract (TOE) on inflammatory markers and cytokines determined in chronic colitis tissues with western blotting. Six animal samples (colon lysates) per group were analyzed in duplicate. Normal, DSS-/D.W; colitis, DSS + /D.W, colitis + 5-ASA, DSS + /5-ASA 50 mg per kg; colitis + TOE, DSS + /TOE 15 mg per kg.

**Figure 8:**
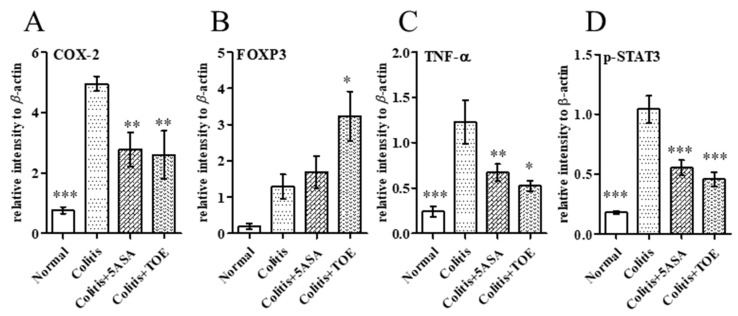
Relative intensity of protein expression levels in chronic colitis tissues of C57BL/6 mice determined using western blotting. (**A**) Cyclooxygenase-2 (COX-2), (**B**) forkhead box P3 (FOXP3), (**C**) tumor necrosis factor alpha (TNF-α), (**D**) phosphorylated signal transducer and activator of transcription-3 (p-STAT3). * *p* < 0.05, ** *p* < 0.01, *** *p* < 0.001, compared with the colitis group. Normal, DSS-/D.W; colitis, DSS + /D.W, colitis + 5-ASA, DSS + /5-ASA 50 mg per kg; colitis + TOE, DSS + /TOE 15 mg per kg.

Regarding neutrophilic activity, all DSS-induced colitis groups showed increased MPO activity in colon tissue lysate. MPO activity of the colitis group (75.00 ± 37.80 µU/mg) recorded the highest value, whereas that of colitis + 5-ASA (38.69 ± 15.95 µU/mg) and colitis + TOE (23.34 ± 6.02 µU/mg) decreased. MPO activity in the normal colon was only 20.79 ± 14.95 µU/mg. These figures were statistically significant (Figure 6E). Exploration of the general inflammatory markers in colitis using western blot, showed that COX-2 and TNF-α expression in both colitis + 5-ASA and colitis + TOE groups was markedly diminished compared to that in the colitis group (Figure 7, Figure 8A,C).

### 2.6. TOE Administration Induced FOXP3+ Treg Cell Response in Chronic Colitis

There were some discrete changes in the regulatory markers of inflammation observed during this study. It was found that a statistically high level of FOXP3 protein was expressed in the colon of the colitis + TOE group (Figure 7 and Figure 8B). We also examined the Th17 reaction of p-STAT3 expression, confirming that FOXP3 overexpression was concomitant with Th17 downregulation. Western blot analysis showed that p-STAT3 expression was significantly decreased in the colon tissues of the colitis + TOE group (Figure 7 and Figure 8D). We also tested for IL-17 and IL-10 antibodies using western blotting, although they were not detected in this study (data not shown).

### 2.7. TOE Administration Altered Th1, Th2, Th17 and Treg Subsets and Cytokine mRNA Expression in Chronic Colitis

**Figure 9:**
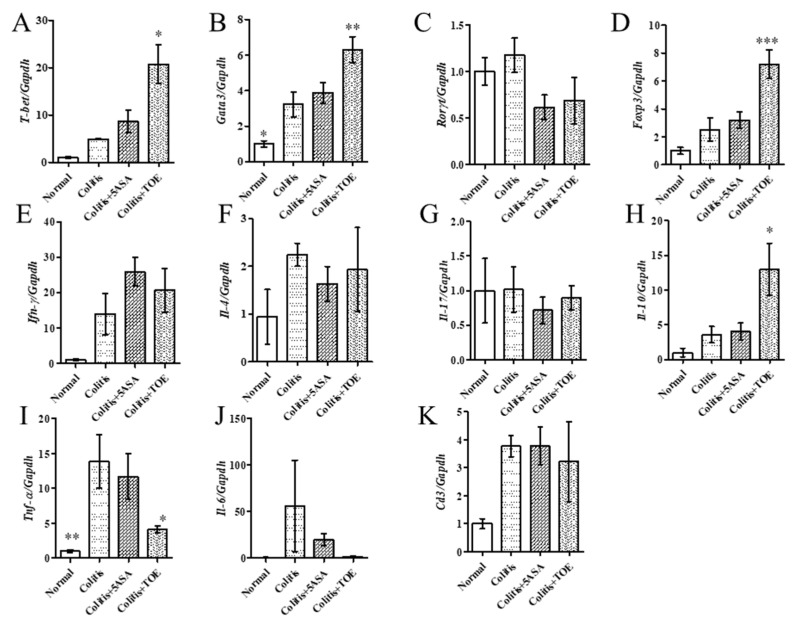
Effect of *Turbinaria ornata* extract (TOE) on helper T cell subsets markers and related cytokine mRNA expression levels in chronic colitis tissues of C57BL/6 mice. (**A**) *T-bet* (**B**) *Gata3* (**C**) *Rorγt* (**D**) *Foxp3* (**E**) *Ifn-γ* (**F**) *Il-4* (**G**) *Il-17* (**H**) *Il-10* (**I**) *Tnf-α* (**J**) *Il-6* (**K**) *Cd3*. * *p* < 0.05, ** *p* < 0.01, *** *p* < 0.001, compared with the colitis group. Normal, DSS-/D.W; colitis, DSS + /D.W, colitis + 5-ASA, DSS + /5-ASA 50 mg per kg; colitis + TOE, DSS + /TOE 15 mg per kg.

After confirming the opposing expression of high FOXP3 and low p-STAT3 in the colon with TOE treatment, the major subsets of helper T cells and related cytokine mRNA expression levels were examined using qPCR. The results showed that Th1 and Th2 transcription factors (*T-bet* and *Gata3*, respectively) were greatly increased; however, related cytokines such as *Ifn-γ* and *Il-4* did not show significant changes after TOE administration (Figure 9A,B,E,F). In contrast, the Th17 transcription factor *Rorγt* and *Il-17* mRNAs were somewhat diminished, although not significantly (Figure 9C,G). Treg specific *Foxp3* and *Il-10* mRNA expression levels were remarkably elevated (Figure 9D,H). *Tnf-α*, which is a cytokine related to both macrophages and the Th1 subset, also exhibited a sharp decline in mRNA level (Figure 9I). These results were comparable with those of the western blot analysis. In addition, *Il-6*, which is another important pro-inflammatory cytokine in IBD, decreased in the colitis + TOE group, although the difference was not significant (Figure 9J). We also examined the pan T cell marker *Cd3* mRNA, which showed no change in all DSS-induced colitis groups (Figure 9K).

## 3. Discussion

*T. ornata* is an edible brown alga (seaweed) that is common worldwide. Research on the anti-inflammatory potential of *T. ornata* has been limited. The inhibitory effect on cotton pellet induced by granuloma was the first report to investigate the anti-inflammatory effect of *T. ornate* [27]. Recently, our research team reported the anti-inflammatory effect of *T. ornata* in an in vitro model of IBD comprising human epithelial Caco-2 and THP-1 macrophages [28]. Sulfoquinovosyl monoacylglycerols and steroids were identified in the extract of *T. ornata*. Among the isolated compounds, sulfoquinovosyl monoacylglycerols that possess carbon chains of C18 (compounds **5** and **7** in this study) were found to be biologically active metabolites that inhibit the expression of inflammatory proteins and nucleic translocation of NF-κB [28]. In the present study, the chemical profile of TOE was deduced through LC-QTOF-MS analysis. It was observed that TOE mainly contained steroids and sulfoquinovosyl monoacylglycerols, which were detected in the positive and negative mode, respectively. We attempted to investigate the in vivo therapeutic effect of TOE, including that of these bioactive metabolites, using an animal model of UC.

First, 6 weeks of TOE treatment (15 mg/kg) in chronic colitis-induced C57BL/6 mice showed marked clinical improvement. Body weight loss in the TOE-treated group was minimal compared with that in the colitis or colitis + 5-ASA groups. The DAI score, the quantified data of clinical manifestation of colitis, was significantly lower in the colitis + TOE group than that in the colitis group. The clinical improvements were also obvious in the results of autopsy and microscopy investigations. Shortening in colon length was protected with TOE administration, and further histopathological evaluation concluded that TOE administration alleviated lesions owing to chronic colitis. These in vivo results strongly suggest that TOE is effective in preventing UC.

To understand how TOE influences the anti-inflammatory effect, the expression of some common pro-inflammatory markers was monitored in the colon tissues. MPO is one of the most abundant peroxidation enzymes in neutrophils, but is less in monocytes [36]. It is released from activated neutrophils into the extracellular matrix and directly causes epithelial death and tissue damage [36]. Thus, measuring MPO activity in colitis tissue is an effective method to estimate the extent of neutrophil infiltration. In this study, TOE-treated colon tissue showed less MPO activity, which means that TOE can directly reduce neutrophilic inflammation in the colonic mucosa.

There is also a potent inflammatory mediator, COX-2, which is induced by several stimuli such as lipopolysaccharide (LPS), TNF-α, and IL-1, and converts arachidonic acid to strong inflammatory mediators such as prostaglandins, prostacyclin, and thromboxane [37]. COX-2 was chosen for this study because it is barely expressed in the normal state but is upregulated in inflamed mucosa and in colitis tissues [37]. In addition, a recent study suggested a new insight into the pathogenesis of UC. At the initiation of colitis, colonic epithelium-specific mammalian target of rapamycin complex 1 induces COX-2 via p-STAT3, which then mediates the Th-17 response [38]. The pro-inflammatory cytokine TNF-α, which is another noticeable target of biological agents of UC such as infliximab, adalimumab, and golimumab was also analyzed [39]. The present study revealed that TOE administration led to a dramatic reduction in both COX-2 and TNF-α expression. These results confirmed that TOE has potent anti-inflammatory effects.

Among the investigated mechanisms of the anti-inflammatory effect of TOE, remarkable changes in CD4^+^ T cell subsets expression levels were observed. In UC, regulation of pro-inflammatory Th1, Th2, and Th17 cells and induction of Treg subsets are the key targets for colitis treatment [40]. Treg subsets are induced by TGF-β, express FOXP3, and produce IL-10 [11,12]. FOXP3 is not only a specific marker of both naturally occurring Tregs and induced Tregs, but is also necessary and sufficient for the immunosuppressive activity of Treg cells [41,42]. Treg cells play important roles in alleviating colitis. It limits both effector T cells and the innate inflammatory response [15]. It plays a pivotal role in the inhibition of a broad range of inflammation. There are four key mechanisms by which Tregs suppress the immune response: (1) secretion of the anti-inflammatory cytokine IL-10 (2) CD28 family (such as (cytotoxic T-lymphocyte-associated protein 4, CTLA-4 and programmed cell death protein 1, PD-1)) and CD25 surface molecule signaling, (3) cytotoxic activity, and (4) metabolic control [43]. In a previous study, IL-10 was demonstrated to be a potent inhibitor of the production of IL-1α, IL-6, IL-8, and TNF-α by human monocytes and macrophages [44]. TOE administration led to a significant increase in FOXP3 and IL-10 expression, which indicates the manifestation of Treg activity.

Among the Janus kinase/STAT pathway molecules, STAT3 is a widely known transducer that produces several pro-inflammatory cytokines; interferons, TNF-α, and IL-6; it is essential for Th17 differentiation, and its activation is closely related to IBD [17]. In the western blot analysis, colons that were treated with TOE showed decreased p-STAT3 expression, whereas FOXP3 expression was increased. This result explains how TOE reduces chronic colitis. In addition, 5-ASA treatment could not induce FOXP3, but down-regulated p-STAT3.

In colitis, continued stimulation of IL-6 activates STAT3 signaling, and is prone to modulating the Th17/Treg immune balance owing to Th17 responses [45]. Therefore, the decrease in both IL-6 and p-STAT3 was additional evidence that TOE is involved in the regulation of immune balance through reducing Th17 activity. Unfortunately, our analysis failed to detect IL-17 and IL-10 proteins, which are effector cytokines of Th17 and Treg, respectively. It is speculated that an insufficient amount of tissues was tested so that the content of these markers might have been under the detection limit of western blotting. These cytokines were detected by qPCR analysis and a significant increase in *Il-10* mRNA expression was observed.

In contrast, some inconsistencies were found regarding our expectations: (1) there was no alteration in *Rorγt* and *Il-17* mRNA expression in the colitis + TOE group, whereas an increment in *Foxp3*, *Il-10*, and reduction in *Il-6* and p-STAT3. At the beginning of this study, we assumed that activation of FOXP3^+^ Tregs leads to Th17 downregulation. These conflicting results may represent the existence of double-expressing cells: RORγT^+^ FOXP3^+^ or IL-17^+^ FOXP3^+^ cells [15]. However, confirmation of the existence of these double positive cells requires performing flow cytometer on colonic lamina propria cells. (2) Interestingly, it is also incomprehensible that the Th1 transcription factor T-bet and Th2 transcription factor *Gata3*, were all significantly increased, whereas the secreted cytokines *Ifn-γ* and *Il-4* did not change or even decrease. These discrepancies are seldom reported; however, it might suggest that the administration of therapeutic agents (such as TOE) could have multifactorial functions. This phenomenon should certainly be investigated in further studies.

In conclusion, TOE is effective in preventing chronic colitis through the upregulation of FOXP3^+^ Treg cells and its secreted anti-inflammatory cytokine, IL-10. It showed a reduction in TNF-α, COX-2, and IL-6 followed by that in p-STAT3 as well as in MPO activity and histological lesions. All these palliative effects were closely related to Treg activation. Although it could not directly detect the IL-10 protein, *Il-10* mRNA expression, and high levels of FOXP3 protein in colitis tissues would support the hypothesis that TOE could induce Tregs. However, further investigation using flow cytometry is needed to confirm the immune cell subpopulations.

## 4. Materials and Methods

### 4.1. Preparation of the Fraction M2 from a Brown Algae T. ornata

A brown algae *T. ornata* was collected at Fulhadhoo in Maldive in August 2017. The research sample was confirmed by Professor You-Jin Jeon at Jeju National University, South Korea. The sample (3.0 Kg) was lyophilized and then was extracted with 90% aqueous EtOH (2 L) twice at room temperature. The extracts were concentrated in vacuo and suspended with water (1.5 L). Following this, the aqueous layer was sequentially partitioned with hexane (1 L), CHCl_3_ (1 L), ethyl acetate (1 L) and BuOH (0.7 L). The BuOH-soluble part (1 g) was subjected to column (3 × 100 cm) chromatography with Sephadex LH-20, eluted with 100% MeOH to yield four fractions (M1~M4). Fraction M2 (230 mg) prepared via elution from 50 to 60 min at a flow rate of 1.0 mL/min showed good activity in NO and IL-10 inhibition assays referred to TOE. TOE was concentrated in vacuo and freeze dried. For administration of TOE to animals, the fraction was weighed accurately and suspended in 0.5%-carboxymethyl cellulose (CMC).

### 4.2. Isolation of Sulfoquinovosylglycerols and Steroids from T. ornata

Bioactive fraction M2 was separated into three major fractions by HPLC using a column YMC ODS-A 250 × 10 mm, isocratic eluting solvent of 95% aq. MeOH including 0.1% formic acid, and refractive index detector. The first fraction (Fr.1, 40 mg) at the retention time of 6.5 min was separated to yield compounds **5** (2.1 mg), **6** (1.2 mg) and **7** (5.4 mg) using gradient HPLC method [column: Phenomenex C8 250 × 4.6 mm, flow rate: 1.0 mL/min, A: H_2_O with 0.1% formic acid, B: ACN, 20% ACN linearly increase to 100% ACN for 20 min, detector: ELSD]. Other two fractions (Fr.2, 15 mg and Fr.3, 80 mg) at a retention time of 19~21 min were separated into a steroid compound **1** (5.0 mg). Further purification by HPLC was conducted by a YMC ODS-A (250 × 10 mm) column eluting 95% aq. ACN solvent.

### 4.3. LC-MS Analysis

A HPLC ExionLC (SCIEX, Framingham, MA, USA) coupled to a quadruple time-of-flight X500R mass spectrometer (SCIEX, Framingham, MA, USA) was used for LC-MS analysis. The sample separation was performed on a Kinetex C18 column (50 mm × 2.1 mm, 2.6 μm) (Phenomenex, Torrance, CA, USA). The HPLC solvents were composed of ultrapure water containing 0.1% (*v*/*v*) formic acid and acetonitrile, indicating solvent A and B, respectively. The two eluting solvents were mixed in a gradient way, which 30% solvent B was linearly increased to 100% solvent B for 15 min with a flow rate of 0.2 mL/min. The electrospray ionization (ESI) source of the MS and MS/MS in positive mode was operated as follows: IonSpray voltage 5500 V; curtain gas 30 psi; ion source gas 1 50 psi; ion source gas 2 50 psi; declustering potential 80 V; temperature 550 °C; collision energy 35 ± 15 V. The mass ranges were *m*/*z* 100~1500 for QTOF MS and *m*/*z* 50~1500 for MS/MS. The MS/MS spectra was obtained by the information-dependent acquisition (IDA). The parameters for the MS and MS/MS spectra in negative mode are nearly same except for IonSpray voltage −4500 V; declustering potential −80 V; collision energy −35 ± 15 V. The calibration of molecular weight was followed by every 5 samples. The acquired MS and MS/MS data were analyzed by SCIEX OS 1.6.1 software (SCIEX, Framingham, MA, USA).

### 4.4. Experimental Animals

Seven-week old female C57BL/6J mice were purchased from Japan SLC, Inc. (Hamamatsu, Japan). All mice were housed in controlled temperature (20~27 °C) and 12 h/12 h light/dark cycle, and standard rodent chow pellets and reverse osmosis filtered water were fed freely. After 2 weeks of acclimation period, animals were randomly divided into six mice per group (*n* = 6) and placed wire cages individually. Animal care and experimental protocols were approved by the Institutional Animal Care and Use Committee at Gyeongnam Department of Environmental and Chemistry, Korea Institute of Toxicity.

### 4.5. DSS-Induced Chronic Colitis and Treatment Protocol

Chronic colitis was induced by three cycles of 1.5% DSS (MP Biomedicals) in drinking water ad libitum for 7 days followed by 7 day of recovery, referred to the previous publication [46]. Normal control group were given tap water without DSS for same experimental period. Samples were administered by oral route once daily. There were 4 groups in this experiments; (i) normal: DSS-/D.W), (ii) colitis: DSS + /D.W), (iii) colitis + 5-ASA: DSS + /5-ASA 50 mg per kg), and (iv) colitis + TOE: DSS + /TOE 15 mg per kg) group (*n* = 6).

### 4.6. Body Weight Change and DAI Score

All animals were carefully monitored its clinical symptoms and weighed every day. For evaluation of DAI, body weight change, stool consistency and blood in feces were observed and scored by minor modification of the previous study [40] as follows; body weight change (no change or gain = 0, ≤ 5% = 1, 6~10% = 2, 11~15% = 3, ≥ 15% = 4), stool consistency (normal = 0, soft but remain formed stool = 1, diarrhea = 2), blood in feces (negative = 0, positive = 2). The DAI was calculated through all experimental day and its changes over time demonstrated by area under the curve (AUC).

### 4.7. Colon Length Measurements and Histopathologic Evaluation

At the end of the treatment, animals were euthanized by CO_2_ chamber and blood was collected immediately from the abdominal aorta. The colon length between the cecum to the anus was measured and dissected longitudinally, three equal strips. One of them was fixed with 10% neutral buffered formalin solution for hematoxylin and eosin (H&E) staining, and others were stored at −72 °C deep freezer for other analysis. Histopathologic examination of colon tissues was performed based on the grading systems of previous publications; inflammatory cells infiltration, ulceration, and mural thickening (0 = none, 1 = mild, 2 = moderate, 3 = severe).

### 4.8. MPO Activity

MPO activity assay was performed with OxiSelect^TM^ myeloperoxidase peroxidation activity assay fluorometric kit (Cell Biolabs, San Diego, CA, USA). Briefly, frozen colon samples were homogenized cold 100 mM phosphate buffer (pH 6.0), containing 0.5% HTAB. Centrifuge the homogenate at 12,000 rpm for 15 min at 4 °C. Then, collect the supernatant and 1:10 diluted with assay buffer, stored on ice. It was prepared that a set of standard, working solution, and MPO peroxidation solution. After loading 50 μL of samples and controls and 50 μL of reagents, immediately read the fluorescence of 544 nm excitation and 590 nm emission filter with microplate reader (Synergy H1, BioTek, Winooski, VT, USA), up to 30 min by kinetic methods. The results were calculated by the datasheet manual.

### 4.9. Western Blot Analysis

Protein extraction from the colon tissue was done by T-PER tissue protein extraction reagent (Thermo Fisher Scientific, Waltham, MA, USA). Equally loaded samples (30 μg) were separated on 10% SDS-PAGE gel and then transferred to the PVDF membrane by iBlotTM-2 transfer kit (Invitrogen, Waltham, MA, USA). Next, membranes were incubated with blocking solution for 1 h and done with primary antibody at 4 °C overnight; TNF-α (Abcam, Cambridge, UK), FOXP3 (Invitrogen, Carlsbad, CA, USA), COX-2 (Abcam, Cambridge, UK), and p-STAT3 (Cell signaling, Danvers, MA, USA) at 1:500 dilution. Washing three times with phosphate buffered saline with Tween 20 (PBST), all membranes were incubated with secondary horseradish peroxidase-linked anti-mouse or and-rabbit for 40 min. After washing step, it was soaked in the enhanced chemiluminescence solution for up to one minute, and its signals were detected by ChemiDoc^TM^ imaging system. Relative density of the bands was analyzed with Image Lab software (Bio-Rad, Berkeley, CA, USA).

### 4.10. RT-qPCR

Total RNA was isolated using QIAzol solution (Qiagen, Hilden, Germany), and then cDNA was synthesize with QuantiTect reverse transcription kit (Qiagen) according to the manufacturer’s guideline. For the real-time PCR, a total of 50 μL reaction mix constituted with 100 nM of cDNA template, 10 pM of each primers, and SYBR green master reagent (Go Taq, Promega, Madison, WI, USA) was prepared. Then, PCR was performed initial denaturation step at 95 °C for 2 min followed by 40 cycles of 95 °C for 15 s and 60 °C for 60 s by the TP 950 thermal cycler (Takara Bio, Shiga, Japan). All samples were analyzed twice. The results were normalized with GAPDH and then calculated by ΔΔCt method. Primer sequences for this experiment were provided in Supplementary Materials S2.

### 4.11. Statistical Analysis

All statistical analysis was performed with SPSS statistics 17.0 program (SPSS Inc., Chicago, IL, USA), one-way ANOVA followed by Dunnett’s post hoc test was for the data satisfied with Levene’s test. Otherwise, non-parametric Kruskal–Wallis test with Dunn’s test was performed. *p* values of <0.05 were considered as significant. The results were converted to the graph by GraphPad Prism 5 (San Diego, CA, USA), which all columns of the graph had error bars denoted standard error of the mean.

## Figures and Tables

**Table 1:** Identification of compounds in M2 fraction of *Turbinaria ornata* extract (TOE) using LC-QTOF-MS analysis.

No	R_t_ (min)	Molecular Formula	*m*/*z* obs.	Error (ppm)	MS/MS Fragments
**1**	12.57	C_27_H_42_O_2_	399.3245 [M+H]^+^	0.6	71.0491, 127.1117, 255.2107, 381.3152
**2**	13.39	C_27_H_42_O	383.3304 [M+H]^+^	0.6	107.0851,109.0648, 133.0648, 147.1777,161.0961, 175.1117
**3**	9.80	C_29_H_46_O_2_	427.3573 [M+H]^+^	1.3	81.0699, 109.0648, 137.0598, 273.1849
**4**	10.13	C_29_H_46_O_3_	443.3519 [M+H]^+^	0.6	81.0699, 109.0648, 161.0961, 267.2475, 315.2682, 425.3414
**5**	6.09	C_27_H_50_O_12_S	597.2954 [M−H]^−^	0.6	225.0056
**6**	9.53	C_25_H_48_O_11_S	555.2843 [M−H]^−^	0.3	225.0075
**7**	13.16	C_27_H_52_O_11_S	583.3154 [M−H]^−^	0.6	225.0056
**8**	8.92	C_18_H_34_O_3_	297.2430 [M−H]^−^	1.7	155.1078, 171.1027, 279.2330
**9**	13.04	C_16_H_32_O_2_	255.2328 [M−H]^−^	0.6	255.2330

## Supplementary Materials

The following are available online at https://www.mdpi.com/2218-273X/10/10/1463/s1, S1: Inhibitory activities of fractions M1~M4 of *T. ornata* extract on nitric oxide and inflammatory cytokines in LPS-activated RAW264.7 cells, S2: Primer sequences for RT-qPCR.

## Abbreviations

IL	Interleukin
COX	Cyclooxygenase
DAI	disease activity index
FOXP3	forkhead box P3
GATA3	GATA binding protein 3
HRESIMS	High Resolution ElectroSpray Ionization Mass Spectrometry
IFN-γ	interferon gamma
LC-QTOF-MS	Liquid Chromatography Time-of-Flight Mass Spectrometry
MPO	Myeloperoxidase
NF-kB	nuclear factor kappa-light-chain-enhancer of activated B cells
Nrf2	nuclear factor erythroid 2 related factor 2
p-STAT3	signal transducer and activator of transcription 3
RORγT	retinoic-acid-receptor-related orphan nuclear receptor gamma
T-bet	T-box expressed in T cells
Th	T helper cells
TNF-α	tumor necrosis factor alpha
Treg	regulatory T cells
