# Supplementary material for: Standardized Fraction of Turbinaria ornata Alleviates Dextran Sulfate Sodium-Induced Chronic Colitis in C57BL/6 Mice via Upregulation of FOXP3+ Regulatory T Cells
Source: Biomolecules. 2020 Oct 20;10(10):1463. doi: 10.3390/biom10101463 (PMC7590162; doi:10.3390/biom10101463)
Supplement: Supplementary file 1 [file biomolecules-10-01463-s001.pdf]

Supplementary Materials

The standardized fraction of *Turbinaria ornata* alleviates dextran sulfate sodium-induced chronic colitis in C57BL/6 mice via upregulation of Foxp3+ T cell response

Na-Hyun Kim[1], Seon Min Lee[1], Yun Na Kim[2], You-Jin Jeon[3], Jeong Doo Heo[1], Eun Ju Jeong[2*] and Jung-Rae Rho[4*]

[1]Gyeongnam Department of Environment & Toxicology, Korea Institute of Toxicology, 17 Jegok-gil, Munsan-eup 5834, Republic of Korea; nhkim@kitox.re.kr (N.-H.K.); smlee84@kitox.re.kr (S.M.L.); jdher@kitox.re.kr (J.D.H.)

[2]Department of Agronomy and Medicinal Plant Resources, Gyeongnam National University of Science and Technology, Jinju 52725, Republic of Korea; skdbassk@hanmail.net (Y.N.K.); ejjeong@gntech.ac.kr (E.J.J.)

[3]Department of Marine Life Sciences, Jehu National University, Jeju 690-756, Republic of Korea; youjin2014@gmail.com

[4]Department of Oceanography, Kunsan National University, Kunsan 54150, Republic of Korea; jrrho@kunsan.ac.kr

*Correspondence: ejjeong@gntech.ac.kr (E.J.J.); jrrho@kunsan.ac.kr (J.-R. R); Tel.: +82-55-751-3224 (E.J.J.); +82-463-69-4606 (J.-R.R.)

S1. Inhibitory activities of fractions M1~M4 of *T. ornata* extract on nitric oxide and inflammatory cytokines in LPS-activated RAW264.7 cells.

Fractions	Conc. (ug/ml)	Relative % of control			
		Nitric Oxide	IL-1β	IL-10	IL-6
M1	20	97.1	99.8	85.5	89.5
M2	20	27.2	99.9	1.10	56.7
M3	20	44.3	88.3	1.12	102.4
M4	20	95.5	98.8	90.0	99.5

S2. Primer sequences for RT-qPCR

TNF-α; 5′-tatggctcagggtccaactc-3′, 5′-ctccctttgcagaactcagg-3′,

IL-17; 5′-ttcagggtcgagaagatgct-3′, 5′-aaacgtgggggtttcttagg-3′,

IL-10; 5′-ccagggagatcctttgatga-3′, 5′-aactggccacagttttcagg-3′,

IL-4; 5′-ggtctcaacccccagctagt-3′, 5′-gccgatgatctctctcaagtgat-3′,

IL-6; 5′-gttctctgggaaatcgtgga-3′, 5′-tgtactccaggtagcta-3′,

IFN-γ; 5′-gcgtcattgaatcacacctg-3′, 5′-tgagctcattgaatgcttgg-3′,

CD3; 5′-atcactctgggcttgctgat-3′, 5′-tagtctgggttgggaacagg-3′,

Foxp3; 5′-tcttgccaagctggaagact-3′, 5′-ggggttcaaggaagaagagg-3′,

RORγt; 5′-gggatccactacggggttat-3′, 5′-gtgcaggagtaggccacatt-3′,

GATA3; 5′-gtcatccctgagccacatct-3′, 5′-agggctctgcctctctaacc-3′,

T-bet; 5′-cctggacccaactgtcaact-3′, 5′-aactgtgttcccgaggtgtc-3′,

GAPDH; 5′-aactttggcattgtggaagg-3′, 5′-ggatgcagggatgatgttct-3′
